# EXAMINING DISCREPANCIES IN SOCIAL ROBOT VERSUS HUMAN ASSESSMENTS OF GERIATRIC WELL-BEING

**DOI:** 10.1093/geroni/igz038.1199

**Published:** 2019-11-08

**Authors:** Erin Harrington, Ha Do, Alex J Bishop, Celinda Reese-Melancon, and Weihua Sheng

**Affiliations:** Oklahoma State University, Stillwater, Oklahoma, United States

## Abstract

Socially assistive robotic (SAR) technologies represent a viable tool for monitoring the safety and health of older adults. However, it is unclear whether SARs can comprehensively screen geriatric well-being as effectively as trained human clinicians. The purpose of this study was to compare SAR versus human assessment of geriatric well-being. Participants included 30 older adults (Mage = 73.40, SD = 7.88) who completed a robot-administered well-being assessment session during which human-administered evaluation was simultaneously performed. Standardized clinical screening assessment tools common in geriatric care were administered (e.g., Short Blessed Test (SBT), UCLA Loneliness Scale, Geriatric Depression Scale, PHQ-4, Iowa Fatigue Scale, Fall Risk). Multiple dependent sample t-tests were used to explore variability in assessment scores between SAR and human evaluation. Assessment scores significantly differed on several measures, including the SBT (t(29) = -9.33, p < .001), UCLA Loneliness scale (t(19) = 2.37, p < . 05), and fall risk assessment (t(29) = 3.03, p < .01). Specifically, the SAR indicated that older adults were significantly more cognitively impaired, less lonely, and more likely to fall compared to the human administrator. Other observed differences and hypothesized explanations will be discussed in greater detail. The current study indicates that there is a divergence in geriatric assessment outcomes based on human versus SAR administration. Findings have implications relative to further developing SAR technology to align with human-based evaluations to enhance cognitive well-being, social connectedness, and falls prevention.

